# The Story of the Insane from Year to Year

**Published:** 1901-03-23

**Authors:** 


### THE STORY OF THE INSANE FROM YEAR TO YEAR.

### THE HULL BOROUGH ASYLUM.

On January 1st, 1899, there were in residence 546 patients, and, practically, the numbers were stationary during the year. The first admissions numbered 178, the readmissions 24, the recoveries 68, the discharged relieved 43, and the deaths 79. The percentage of recoveries stands at 35 on the admissions of the year, and the deaths at 14·26 on the average number resident. Of the deaths 45 were caused by cerebral and spinal diseases, 12 by phthisis, 3 by pneumonia, and 2 by enteritis. Of the admissions 6 are supposed to have owed their insanity to various moral causes, 33 to intemperance in drink, 35 to previous attacks, 18 to hereditary influence and in 48 instances the cause was unknown.

Dr. Merson's report invariably well repays perusal. When considering the mortality from tuberculous disease he points out that, in his asylum, it has increased from 16 per 1,000 to 24 per 1,000; and although he cannot offer any satisfactory explanation of this fact, he says it "suggests the necessity of increased care in the disinfection and isolation of such cases, as well as of attention to the purity of the milk supply." On that indisputable and sad fact, the increase of the certified insane, Dr. Merson points out to his committee that at the present rate of increase, "all the available space will be used up in a little over five years." It may readily be understood that the committee will dislike the idea of again enlarging the asylum; but it is the only apparent course consistent with economy and convenience, and we hope the work will be speedily taken in hand. The committee again express their "pleasure with the satisfactory manner the patients have been attended to." The weekly cost of maintenance is 9s. 7d. per head.

### THE MIDDLESEX COUNTY ASYLUM AT WANDSWORTH.

This asylum contained on January 1st, 1899, 1,384 patients, and that number had risen to 1,415 at the end of December. There were 347 first admissions, 51 readmissions, 125 recoveries, 44 discharged relieved, and 133 deaths. Calculated in the way usual in asylums the recoveries stand at 34·9 per cent., and the deaths at 9·4 per cent. The average percentage of recoveries at Wandsworth for the last sixty years was 39·6, and the average death rate for the same period was 8·8 per cent. Of the deaths during the last year 65 were caused by cerebral and spinal disease, 13 from phthisis, 4 rom bronchitis, and 1 from pneumonia. Of the year's admissions 73 owed their insanity to moral causes, including domestic trouble, adverse circumstances, religion, &c.; and of the physical causes drink is responsible for 26, hereditary influence for 56, congenital defect for 35, and in 95 no cause was ascertained. Like so many others this asylum is full, and 166 lunatics chargeable to Middlesex are boarded out in other asylums. It would seem from Dr. Hill's report that the Middlesex Asylum patients increase by about 73 every year, and until the new asylum at Napsbury is opened there is sure to be much difficulty in finding accommodation for the annual increment. Even when this accommodation can be obtained it is obtained at greater cost to the ratepayers, both direct and indirect. Direct because a higher sum is charged for maintenance of those boarded out, and indirect because the absence of the quiet, harmless, working patients, and the presence of acute patients, must necessarily raise the maintenance rate in the asylum. In view of these facts, and many others, it is to us a never-dying source of wonder why County Councils so often put off building operations. The visitors say that Dr. Hill "continues to devote his energy and zeal to the successful administration of the asylum." There are now four assistant medical officers. The weekly charge to the unions is 11s. 6d. per head.

### THE CHESHIRE COUNTY ASYLUM AT MACCLESFIELD.

There were 726 patients in residence on January 1st, 1899, and only 723 on December 31st. The first admissions were in number 145, the readmissions 46, the recoveries 54, the relieved 12, the deaths 64. The recoveries were 28·8 per cent. of the admissions, and the deaths were 8·9 per cent. of the average number resident. Of the year's deaths 22 were caused by cerebral and spinal diseases, 8 by pneumonia, and 16 by phthisis. Table X. shows us that 40 of the year's admissions owed their insanity to moral causes, 42 to intemperance in drink, 42 to previous attacks, and 21 to hereditary influence. The asylum is rather more than full, and it is proposed to spend £70,000 in enlargements and improvements. The committee have during the year paid over to the County Council small sums recovered as profits from the out-county, county, and private patients, and £1,000 from the maintenance account. The two first payments are certainly correctly made; but as the repairs to the asylum will cost considerably more than £314, it seems a strange proceeding to pay money over to the county treasurer and draw it back again. Regarding the £1,000 it has been saved from the sums paid by the unions, and it is now handed over to the county. It would have been better to reduce the maintenance charge and draw on the maintenance balance. By this means each union would get its money back again in something like the same proportion it parted with it; but how the County Council can divide it to the unions does not seem very clear. The recovery rate was low; but a high rate could not be expected considering the material; for we hear that 23 of the admissions were imbecile, 23 were epileptic, 47 were almost in a state of chronic insanity, and in only one-fourth had the disease existed for less than three months. "The committee are glad to be able to again express their satisfaction with the manner in which the asylum is managed by Dr. Sheldon and the other officers." The weekly charge to the unions was 8s. 5½d. per head.

